# Propranolol: a new pharmacologic approach to counter retinopathy of prematurity progression

**DOI:** 10.3389/fped.2024.1322783

**Published:** 2024-01-16

**Authors:** Francesca Pascarella, Rosa Teresa Scaramuzzo, Alessandro Pini, Maurizio Cammalleri, Paola Bagnoli, Massimiliano Ciantelli, Luca Filippi

**Affiliations:** ^1^ Neonatology Unit, Azienda Ospedaliero-Universitaria Pisana, Pisa, Italy; ^2^ Department of Experimental and Clinical Medicine, University of Florence, Florence, Italy; ^3^ Unit of General Physiology, Department of Biology, University of Pisa, Pisa, Italy; ^4^ Department of Clinical and Experimental Medicine, University of Pisa, Pisa, Italy

**Keywords:** proliferative retinopathy, oxygen, beta blockers, angiogenesis, vascularization

## Abstract

Despite the evident progress in neonatal medicine, retinopathy of prematurity (ROP) remains a serious threat to the vision of premature infants, due to a still partial understanding of the mechanisms underlying the development of this disease and the lack of drugs capable of arresting its progression. Although ROP is a multifactorial disease, retinal vascularization is strictly dependent on oxygen concentration. The exposition of the retina of a preterm newborn, still incompletely vascularized, to an atmosphere relatively hyperoxic, as the extrauterine environment, induces the downregulation of proangiogenic factors and therefore the interruption of vascularization (first ischemic phase of ROP). However, over the following weeks, the growing metabolic requirement of this ischemic retina produces a progressive hypoxia that specularly promotes the surge of proangiogenic factors, finally leading to proliferative retinopathy (second proliferative phase of ROP). The demonstration that the noradrenergic system is actively involved in the coupling between hypoxia and the induction of vasculogenesis paved the way for a pharmacologic intervention aimed at counteracting the interaction of noradrenaline with specific receptors and consequently the progression of ROP. A similar trend has been observed in infantile hemangiomas, the most common vascular lesion of childhood induced by pre-existing hypoxia, which shares similar characteristics with ROP. The fact that propranolol, an unselective antagonist of β1/2 adrenoceptors, counteracts the growth of infantile hemangiomas, suggested the idea of testing the efficacy of propranolol in infants with ROP. From preclinical studies, ongoing clinical trials demonstrated that topical administration of propranolol likely represents the optimal approach to reconcile its efficacy and maximum safety. Given the strict relationship between vessels and neurons, recovering retinal vascularization with propranolol may add further efficacy to prevent retinal dysfunction. In conclusion, the strategy of contrasting precociously the progression of the disease appears to be more advantageous than the current wait-and-see therapeutic approach, which instead is mainly focused on avoiding retinal detachment.

## 1 Introduction

Premature infants often face serious health problems, especially when they are born very early. These problems often vary. But the earlier a baby is born, the higher the risk of health challenges. Retinopathy of prematurity (ROP) is a vasoproliferative disorder related to preterm birth and represents one of the complications with the most dramatic outcomes. ROP and infantile hemangiomas (IH) are frequently associated (1, 2) and share many features in common, the main one being the common induction of hypoxia-induced neovascularization (3).

Infantile hemangiomas are typical lesions of infancy, developing between the 2nd and the 4th week of life, affecting 5%–10% of all infants. They are benign proliferative lesions made of a disorganized vascular network, whose main cellular elements are represented by hemangioma stem cells (HemSCs) giving the origin to hemangioma perivascular cells and hemangioma endothelial cells (3). The natural history of these lesions consists of three phases: a proliferative one, lasting till the 5th month, an early involuting phase characterized by a slowing down in the tumor growth, lasting till the end of the 1st year of life, then an eventual late involuting phase characterized by lesion regression (4, 5). These lesions are more common in females (3:1) and premature infants. Other risk factors associated with IH are low birth weight, multiple gestation, advanced maternal age, pre-eclampsia, and other placental abnormalities (6, 7). It's easy to notice that many of these are associated with ROP as well and the two pathologies often co-occur (2) (especially with advanced stages of ROP) (8), hence we can postulate they may have a common underlying pathogenesis. Indeed, they are both vasoproliferative lesions driven by hypoxia and many findings support this statement.

The higher incidence of IHs in newborns with low birth weight due to placental insufficiency and their strong correlation with placental anomalies responsible of disturbances for the utero-placental circulation (9) suggest that these lesions are intrinsically hypoxic and that IHs may represent an attempt to revascularization of relatively hypoxic tissue areas (10). Histological analysis showed that both endothelia of IHs and retinal neovasculature in ROP share the expression of GLUT1 (11), a factor significantly up-regulated in hypoxic tissues and stimulated by hypoxia-inducible factor 1 (HIF-1*α*) (12). ROP angiogenesis is driven by high levels of proangiogenic factors such as Vascular Endothelial Growth Factor (VEGF) (13) or matrix metalloproteinases (MMPs) (enzymes that proteolytically degrade the extracellular matrix and promote angiogenesis) (14), and children with proliferative IHs show serum VEGF concentrations and urine levels of MMP-9 significantly higher than patients with involuting hemangiomas (15, 16). The area of pallor and discoloration seen in the skin area preceding hemangioma formation suggests local hypoxia (10). Furthermore, the aforementioned risk factors are likely to induce hypoxia. Premature birth alters the physiological balance between oxygen tension and vasculogenesis, leading to abnormal vascular development with females more sensitive to hypoxia because of their levels of estrogens that have a boosting action on hypoxia-induced proliferation of Endothelial Progenitor Cells (EPCs) (17).

In premature infants, ROP occurs when blood vessels swell and grow too much in the light-sensing tissue at the back of the eye, called the retina. Sometimes these overgrown vessels slowly scar the retina and pull it out of place. When the retina is pulled away from the back of the eye, it's called retinal detachment. Without treatment, this can harm vision and cause blindness.

Both IH and ROP are multifactorial diseases whose pathogenesis can be represented by an epidemiological triad model (18). The triad shows the interaction between a susceptible host (premature infant with very low birth weight) and the agent (oxygen), both acting in a non-favorable environment (hospital services with poor neonatal care, no screening program, and lack of awareness in parents and health providers, no adequate treatment) (19).

With premature birth, indeed, the newborn is subjected to an important change in environmental exposure before he is physiologically ready. During intrauterine life, the embryo and fetus develop in a low-oxygen environment, although oxygen levels vary across gestation (20). This physiological hypoxia is extremely important to maintain activated HIF-1*α* and its downstream factors, above all VEGF, essential for the development of a physiological vascularization. Furthermore, the placenta is an important source of cytokines and growth factors, such as insulin-like growth factor (IGF-1), which plays an important role in maintaining endothelial cell survival and promoting VEGF-induced vascularization (21, 22). Premature birth causes on one hand precocious exposure to an elevated oxygen tension. This induces increased hydroxylation and degradation of HIF-1α, hence a sudden reduction in VEGF production. On the other hand, loss of the placenta causes a severe reduction of serum levels of IGF-1. These events, taken together, alter normal retinal vascular development inducing vascular regression. Furthermore, other factors may play a role in the development of the disease, by inducing inflammation and metabolic stress, thus interfering with physiological vasoproliferation. These factors are represented by intrauterine inflammation (like chorioamnionitis), additional perinatal comorbidities including bacterial and fungal infections leading to sepsis (23), and necrotizing enterocolitis (24), especially recurrent in premature newborns.

## 2 ROP pathogenesis

In the human retina, vasculogenesis begins at around the 14th–15th week of gestation, when spindle-shaped endothelial precursor cells start migrating from the optic disk toward the ora serrata; this process is made possible by a tight interaction and cooperation between astrocytes and EPCs (25, 26). The formers precede the front of vascularization and, in response to hypoxia, activate HIF-1α and then produce VEGF thus stimulating the recruitment and the proliferation of EPCs (26); subsequently, early networks of capillaries start forming (25). However, while EPC recruitment and expansion need very low levels of oxygen, the differentiation of EPCs into endothelial cells is strictly dependent on an increase in oxygen levels (27). Therefore, only the EPCs closest to the pre-existing vessels, i.e., exposed to higher oxygen levels, can differentiate, and this process ensures a centrifugal progression of the newly formed capillary network reaching the retinal periphery nasally at 36 weeks of gestation and temporally at around 40 weeks of gestation (28, 29).

Furthermore, the slight but progressive increase in oxygen partial pressure starting from around the 33–34th week of gestation may additionally accelerate retinal vascularization (20, 30), which is completed at around 38–40th week of gestation (31).

Premature birth causes a blockade of this physiological process. Exposure to atmospheric oxygen levels and loss of placenta before the retinal vascular network has fully developed induce interruption of EPC recruitment and sudden differentiation of recruited precursor cells in terminal differentiated cells. The result is a vascular network that would not work properly, with stunted and obliterated vessels (26), unable to provide enough oxygen to the surrounding structures, especially in the peripheric area, leading to ischemia (ischemic phase of ROP).

The role of oxygen in this phase is highlighted by several clinical trials demonstrating that liberal use of oxygen in preterm newborns increases the risk of ROP, while limiting oxygen exposure significantly decreases the risk of developing ROP, although reducing their survival rate (32). Therefore, preterm newborns seem to have a paradoxical relationship with oxygen, which on the one hand guarantees and improves their survival, but on the other hand represents the signal that interrupts the vasculogenic processes and therefore lays the foundations for the development of ROP.

Hyperoxia-induced blockade of retinal vascularization determines a progressive imbalance between the metabolic demand of the developing neural retina and the insufficient supply coming from the poorly developed capillary network thus producing an increasing retinal hypoxia. This leads to the re-activation of HIF-1α and its downstream growth factors such as IGF-1 and VEGF involved in the angiogenic process. Consequently, an abrupt and tumultuous neoangiogenesis develops (proliferative phase of ROP) and it usually takes place between the 32nd and 34th week of postmenstrual age (33). New vessels, intended to connect the avascular peripheric retina with the central zone, are, however, cluttered and disorganized, with many leaking points, altered tight junctions, and blood-retinal barrier (BRB) breakdown (34, 35). This causes protein leakage, edema, and inflammation. As a result, in the advanced stages of the disease, this process can lead to the formation of fibrotic tissue and invasion of the vitreous and lens, thus exposing the retina to a high risk of detachment (26). The evolution towards this stage depends on the degree of ischemia leading to hypoxia, so, in a situation of milder ischemia, there could be an evolution toward normal vascularization (36).

Therefore, while physiologically fluctuating hypoxia represents the necessary condition for harmonious retinal vascular development during intrauterine life, abrupt changes in oxygen levels secondary to premature birth can be responsible for deviation from the physiological processes.

Although ROP has been classically described as a vasoproliferative disorder, several findings suggest that it should be considered rather as a neurovascular disease (37). Visual impairments or even visual loss observed in preterm infants with previous ROP have been generally attributed to macular injury and other ocular complications (such as retinal detachment, and glaucoma) resulting from fibrovascular changes. Further studies show an association between ROP and poor neurodevelopmental outcomes as well, especially when considering the most severe form (38). A study performed in North Korea (39) showed a significant association between severe ROP and a higher incidence of neurodevelopmental disorders both in the first year of life and after 10 years, and this association was stronger when compared to mild ROP, although poor neurodevelopmental outcomes may be, *per se*, a complication of preterm birth (40, 41). On the other hand, several follow-up studies show the association between ROP and reduced visual acuity (intended as the capacity of our brain to differentiate contrast variation) (42, 43) even when vascular alterations have regressed, and objective retinal injuries are absent.

Unquestionably, the retina is a neurovascular structure: the vascular plexuses are in close contact with neural ganglion cells (44), which start in the retinal neural layers entering then, via the optic nerve, the brain, that's why we may refer to the retina as a “window” to the brain (45). Since ROP is a disorder of the developing retina, it's quite reasonable to state that it affects the neural retina as well: the vascular and the neural retina are exposed to the same growth factors, proliferating and differentiating in close contact with each other and influencing themselves. Indeed, associations between severe ROP and altered functioning of rods and cones, as well as degeneration of retinal ganglion cells, bipolar and amacrine cells, and decrement of their density have been reported in several studies (37). Furthermore, since some of the growth factors involved in the development of the retina such as VEGF and IGF-1 act as well on the developing brain, their dysregulation seen in ROP may affect the development of some cerebral regions, determining injury to both the visual axis and other cerebral areas involving motor and cognitive skills. Supporting this hypothesis, a study showed an association between treatment with bevacizumab (an anti-VEGF agent used for the treatment of advanced stage of ROP) and neurodevelopmental delay in those infants (46), because of VEGF levels reduction in the retina and the brain as well. Moreover, in literature there are many studies showing an association between ROP and smaller cerebellum, brainstem, and greater unmyelinated white matter volume; another prospective study applying Magnetic Resonance Imaging of cerebral white matter (47) found an association between severe ROP and lower fractional anisotropy (meaning reduced white matter integrity) in the posterior white matter, decreased maturation of the optic radiation and the posterior limbs of the internal and external capsule. We can argue that these regions may be peculiarly vulnerable and then susceptible to the same biochemical changes happening in the retina.

But, besides these aspects, it is worth considering that visual impairment, whatever its origin is, is strictly connected to a certain grade of neurodevelopmental impairment, because of a limitation in acquiring cognitive, behavioral, and neurological abilities. Reduced visual acuity originating from ROP may result in pluridisabilities due to difficulties in creating brain connections deriving from a great integration of all sensorial information. So, it remains unidentified whether neurodevelopmental disabilities associated with ROP are secondary to dysregulation of the angiogenic pathway in other areas of the brain or to visual impairment caused by ROP; further studies are needed to clarify these aspects.

Nevertheless, considering this new perspective on the disease, it becomes more and more compelling to find instruments for prevention and treatment before it gets to the advanced stages.

## 3 ROP incidence

ROP represents a leading cause of childhood blindness worldwide (48), especially in developing countries such as India, China, Eastern Europe, and Latin America (49, 50). It is still a big challenge trying to obtain epidemiological indicators of ROP from hospital or population-based studies because of substantial variability in screening criteria and study designs (19). Historically, ROP and ROP-induced blindness have gone through three epidemics. The first one occurred in the decade between 1940 and 1950, because of unmonitored oxygen supplementation to premature newborns and it involved babies weighing around 1,350–1,370 g. It later subsided thanks to a more balanced use of oxygen in neonatal intensive care (51–53). The second one began in the late 60s and early 70s when the advancements in neonatal care increased the survival rate of very low birth weight and extremely premature infants (54–56). Since the early 90s (57), then, we've been going through a third epidemic phase of ROP involving middle- and low-income countries (MIC and LIC). The reason for this 3rd epidemic lies in the increasing survival rate of preterm newborns in MIC and LIC because of a great improvement in supportive and therapeutic services, albeit with suboptimal care, thus exposing more mature infants at risk. Indeed, these centers lack high-quality ROP care—i.e., relevant ROP screening guidelines or policies resulting in the use of unmonitored oxygen, adequate screening programs for early detection of ROP, etc.—facilitating thereby the widespread occurrence of ROP across the developing world (50, 54, 58–60).

The incidence of ROP is variable but is around 60% in the very low birth weight (VLBW) infants. The Early Treatment for Retinopathy of Prematurity (ET-ROP) study conducted in the US showed an incidence of 68% among infants weighing <1,251 g of any stage of ROP (49, 61, 62). On the other hand, in MIC and LIC (such as India, Eastern Europe, and Latin America) high incidence of severe ROP has been reported in newborns weighing more than 1,500 g and gestational age greater than 34 weeks (49, 53). Indeed, in a publication from India, 45% of preterm infants weighing more than 1,250 g developed severe retinopathy (63).

In a global estimation in 2010, 184,700 babies of 14.9 million preterm babies developed any stage of ROP, 20,000 of whom became blind (visual acuity <20/400) or severely visually impaired (visual acuity from <20/200 to ≥20/400) from ROP, and of whom 12,300 others developed mild-moderate visual impairment (visual acuity from <20/40 to ≥20/200) (59). The level of visual impairment due to ROP is also different between high- and low-income countries: in Asia ROP is the first cause of childhood avoidable blindness and the rates of visual loss from ROP are twice as high per million of newborns diagnosed with ROP as what estimated in high-income countries (60). This is the obvious consequence of what was stated before. Indeed, whereas in the West there has been a risk factor transition from first to second epidemic (i.e., from preterm to extreme preterm or very low birth weight), the third epidemic occurring in LIC and MIC is coming from mixed risk factors (19, 64).

Therefore, it becomes more and more evident the need for new treatment strategies which should be easily available and safe to administer, in order to counteract the wide spreading of the disease, its progression, and its long-term consequences.

## 4 Preventive or therapeutic strategies

Prevention of ROP can be developed on three levels:

• Primary prevention (prevention of ROP developing): this acts on ROP-associated risk factors. It is about reducing preterm birth (so prevention of teenage and advanced age pregnancies, avoidance of smoking, substance/alcohol abuse), reducing supplemental oxygen administration during intensive care recovery (even if the ideal range of oxygen saturation target remains controversial), advancement in neonatal care, prevention and precocious treatment of neonatal comorbidities (19, 65).
• Secondary prevention (prevention of ROP-related outcomes): this includes early screening and detection of ROP followed by treatment. This implies having a well-defined screening program, with international guidelines and trained ophthalmologists.
• Tertiary prevention (prevention of further complications of the disease): this includes all the interventions meant to restore the vision and treat the complications (19).

All current treatment strategies may be considered as strategies of tertiary prevention. These strategies, indeed, are aimed at minimizing the effects of disease progression in the advanced stages. They include cryotherapy, then replaced by the more effective and less painful laser photocoagulation, and therapy with anti-VEGF drugs. These therapies are mainly used for the treatment of threshold retinopathy (66–68), showing an efficacy of approximately 90% in avoiding disease progression. As described in the ETROP study, laser treatment has shown great efficacy in the treatment of type 1 ROP (which is the type of ROP with significant changes and thus requiring treatment) (69), although disappointing results are seen in the treatment of Aggressive Posterior-ROP and zone 1 ROP. Despite being a successful first-line treatment for several years, its use is not free from adverse effects and complications. Above all, it requires anesthesia and advanced ophthalmologist skills, and, depending on these ones, around 10% of cases treated with laser photocoagulation require a second intervention (70). In addition, laser treatment is associated with several visual complications: vitreous hemorrhage, hyphema, cataract, increase or decrease in intraocular pressure, increase in refractive errors but, above all, limited field of vision because of disruption of the peripheral avascular retina (57, 36, 71). Further progression in ROP treatment has been made with anti-VEGF use, in order to reduce or avoid complications and sequelae related to the use of laser therapy. Anti-VEGF drugs work to restore the physiological levels of VEGF (which is pathologically increased instead). This way they can prevent further progression of the disease and induce regression of new vessels, showing an acceptable safety profile at 24 weeks after the beginning of treatment (33). The most commonly anti-VEGF agent used in preterm infants is bevacizumab and it has great evidence of successful outcomes for the treatment of threshold ROP either as a single treatment or as an adjuvant to standard laser therapy (66, 67, 72). Indeed, they showed similar effectiveness of laser photocoagulation but with a significantly lower percentage of later refractive alterations (73, 74). On the other hand, the use of anti-VEGF agents is related to several limitations as well. First, they have to be administered by intravitreal injection, hence they require antibiotic prophylaxis and the presence of a trained ophthalmologist. In addition, they can present, even if to a lesser extent, some of the complications seen for laser photocoagulation and a certain incidence of needing retreatment (75). However, the most important potential complication is the transient reduction of serum VEGF level (whose degree varies depending on the anti-VEGF agent used), and this may alter the physiological development of organs such as the brain, lungs, heart, and kidneys. Indeed, there are some studies describing neurodevelopmental outcomes in infants treated with bevacizumab and the results are contrasting. A retrospective study from the Canadian Neonatal network showed more severe neurodevelopmental disabilities in children treated with bevacizumab than in those treated with laser photocoagulation, with the limitation that the study was not randomized, so the more severe outcome could be related either to the more severe form of ROP (76). On the other hand, some studies are showing no significant association (77). Then, more data are needed to study the long-term effects of anti-VEGF agents and many questions about the use of anti-VEGF drugs remain unanswered; moreover, they're still a strategy that cannot prevent vessel neoformation and can only be used for the treatment of threshold disease.

## 5 Propranolol in infants

Over the last decade, the similarities between IH and ROP have become progressively more evident. For both, hypoxia has been demonstrated to be the main promoting trigger that activates the β-adrenergic system. The release of catecholamines and their interaction with β-adrenoceptors (β-ARs) induce the activation of pro-angiogenic and pro-inflammatory factors which determine pathological vascularity. Therefore, the idea of using propranolol for ROP treatment came from the successful use of this drug in inducing regression of IHs. A lot of similarities have been found between these two diseases, and these could help figure out the molecular pathways involved in the pathogenetic processes. In recent years, great advantages are coming from ROP treatment with propranolol. Indeed, it is the first drug that can be used for the treatment of prethreshold disease, thereby preventing vessel tufts neoformation and slowing down the natural history of the disease, reducing the need for a more invasive therapeutic approach. Beyond these aspects, it's worth remembering that it's easily available and, given its historical use in the cardiovascular field, its safety profile has been well studied, even if its kinetic and its effects in the newborn may be somehow different and its mechanism of action in the vasoproliferative lesions has yet to be completely understood.

## 6 Propranolol in the treatment of infantile hemangiomas: its exploitation to the OIR model

The use of propranolol for the treatment of infantile hemangioma became by serendipity. In 2008 Léauté-Labrèze et al. first reported hemangioma regression in eleven children receiving oral propranolol to manage the cardiovascular complications coming from the use of corticosteroids previously used to treat the hemangioma (78). Since this time, subsequent studies have shown the efficacy and safety of propranolol at 1–3 mg/kg per day in the treatment of infantile hemangiomas (79, 80). The well-documented efficacy of propranolol in inducing IH regression aroused the idea of a relevant involvement of the β-adrenergic system in the angiogenetic process. Indeed, angiogenesis is led by hypoxia, and tissue hypoxia is related to an increased norepinephrine release (81), hence hypoxic lesions as IH and ROP are associated with a catecholaminergic overstimulation which is likely to induce a β-AR mediated increase in VEGF expression (82). Indeed, there is a wide expression of β-ARs both in IH cells and in retinal cells. In IH, β1- and β2-ARs are expressed in both hemangioma endothelial cells and stem cells. In the retina, β2-ARs are localized into several retinal cells, including Muller cells (83), while β1-ARs are mainly localized in the inner capillary network, on the surface of endothelial cells (84). Catecholamines are synthesized by sympathetic neurons, and released at the sympathetic terminals, but it is known that under normoxia endothelial cells can synthesize noradrenaline as well (85), implementing its synthesis and release in response to hypoxia. High levels of enzymes involved in catecholamine synthesis are documented in endothelial cells (85) and more specifically in hemangioma endothelial cells (86); in addition, the expression of these enzymes was reduced after treatment with propranolol in IH (86).

## 7 Propranolol in mice with oxygen induced retinopathy

The most widely used model for studying human vasoproliferative retinopathy, such as ROP, and their antiangiogenic treatment is the mouse model of Oxygen-Induced Retinopathy (OIR). This model replicates the pathogenic events leading to the disease. First of all, the rodent retina represents a great model for premature newborn retina, since when rodents are born, their retina is avascular. Its vascularization starts at postnatal day 4, becoming complete during the first week of postnatal life (87) or some days later (88), depending on the strain of mice evaluated. Interestingly, this process is associated with exposure to the higher oxygen level of the extrauterine environment, and reduction of HIF-1 and VEGF levels (89).

Furthermore, the developmental stage of the retinal vascular network of mice during extrauterine life development is quite similar to what happens in the retina of human preterm neonates. Those aspects make the mouse a recommendable model for reproducing ROP.

Since ROP derives from premature exposure of the human retina to high levels of oxygen, a typical OIR model is produced by exposing mice pups for 5 days [between postnatal day 7 (PD7) and PD12] to high oxygen concentrations (75% +/− 2%) (90). This experimental procedure induces an initial response of vessel vasoconstriction and vascular regression in the central zone of the retina, while the peripheric retina is quite spared. Vessel loss in the central retina causes ischemia and hypoxia which, once the mouse is back to room air at PD13 (which is now a “relative hypoxia”), induces expression of proangiogenic factors and new vessel formation, reproducing, then, the proliferative phase of ROP. During the proliferative phase, in the OIR retina, HIF-1α levels are significantly increased together with all its downstream effectors, such as VEGF, VEGFR1, VEGFR2, IGF-1, and IGF-1R messenger (91). Secondary to these molecular changes, a marked vascularization can be seen at the border between the central avascular and the peripheral vascularized retina, and with the formation of engorged vessel tufts expanding into the vitreous, likely to cause retinal hemorrhages (83); retinal neovascularization is maximal at PD17 (92).

In the studies performed by Ristori et al. propranolol was administered to OIR mice subcutaneously three times a day from PD12 to PD16 (83), during the proliferative phase. Systemic administration of propranolol was associated with a reduction of retinal levels of VEGF and IGF-1, thus decreasing retinal neovascularization and vascular leakage by restoring the levels of the tight junction protein occludin and decreasing the extravascular leaking of albumin (83). Interestingly, propranolol did not modify VEGF concentrations in the brain, lungs, or heart, confirming that propranolol downregulated only the levels of VEGF and IGF-1 stimulated by hypoxia. A following study by Dal Monte et al. showed that in the OIR retina, norepinephrine levels increased during the proliferative phase by approximately 90% suggesting that increased levels of noradrenaline may, in turn, overstimulate β-ARs and potentially activate signaling pathways by acting as a proangiogenic switch (83). Among the β-ARs, the finding that the selective antagonism of β2-AR was able to counteract the pathological vascularization, suggested a key role of the axis norepinephrine/ β2-AR for the development of the hypoxia-induce neovascularization (93).

To minimize the side effects of systemic exposure to propranolol, in OIR mice eye drops at 2% concentration resulted in a retinal concentration of the drug similar to what was found after 20 mg/kg/day systemic administration of the drug, with a similar antiangiogenic effect (94). The high lipophilicity of propranolol, together with the presence of hyaluronic acid in the eye drops favored the drug diffusion across the physiological barriers. A following preclinical study conducted on healthy rabbits using propranolol eye drops 0.1% showed an increased retina/plasma ratio of propranolol concentrations compared to 1 mg/kg/day oral administration, suggesting that topical administration is feasible and convenient (95). However, the benefits of propranolol treatment are not limited to its antiangiogenic effects. The study by Martini et al. (93) showed that the selective antagonism of β2-ARs in OIR mice not only reduced the tumultuous vascularization but also improved retinal function, as demonstrated by electroretinography. A similar effect was described some years later by Cammalleri et al. administering propranolol to OIR mice (96). More recently, Qadri et al., demonstrated that propranolol prevented retinal astrocyte degeneration evoking an indirect neuroprotective effect if one considers the relevant role played by astrocytes in retinal function (97).

These intriguing results on the OIR model paved the way to test the safety and the efficacy of propranolol in human preterm newborns with ROP, with the hypothesis that propranolol, a well-tolerated, non-selective β-blocker, would be able to reduce disease progression when administered in a precocious phase of the disease.

## 8 Propranolol in humans with retinopathy of prematurity

Based on these promising preclinical results, a series of clinical trials have been carried out testing oral propranolol in very low birth weight extremely premature newborns with ROP. Propranolol was administered, at newborns at stage 2 ROP, at doses up to 2 mg/Kg/day, the dose used for infantile hemangioma (98–106). As demonstrated in four recent meta-analyses, treatment with oral propranolol minimizes the progression of the disease reducing the need for anti-VEGF administration and laser (107–110). VEGF plasma levels remained unchanged in patients treated with propranolol (98) confirming what was seen in the OIR mice model, i.e., that propranolol antagonizes selectively the VEGF produced in the hypoxic retina without modifying VEGF produced in other normoxic districts. This is a great advantage when compared to anti-VEGF drugs, whose main concern was indeed related to its possible systemic effect.

The main concerns about this treatment, however, refer to its safety because the systemic intake of an unselective β-blocker has potential risks. Stable newborns definitely well tolerated treatment with propranolol (stability of biochemical, hemodynamic, and respiratory parameters), but serious side effects were seen in patients with unstable clinical conditions who showed cardiorespiratory complications such as bradycardia, bradi/apnea, and hypotension, which required treatment interruption.

Given the safety concerns arising by the systemic administration, topical administration through eye micro-drops was explored in the retina of preterm newborns. Two clinical trials were carried out by Filippi et al. with propranolol 0.1% eye micro-drops showing a safety profile but without efficacy on ROP progression (111) and with propranolol 0.2% eye micro-drops that maintained the safety profile but displayed a positive effect in arresting ROP progression (112). While in the study with propranolol 0.1% the administration of eye micro-drops was begun in neonates with ROP stage 2, in the study with propranolol 0.2% the treatment was planned at ROP stage 1, following the idea that a more precocious treatment could be more effective. During this second study, the observation that newborns treated (for cardiac indications) with propranolol before ROP development showed a ROP more aggressive, suggested that propranolol was effective not as a primary preventive treatment but only if administered during the proliferative phase (112).

**Figure 1:**
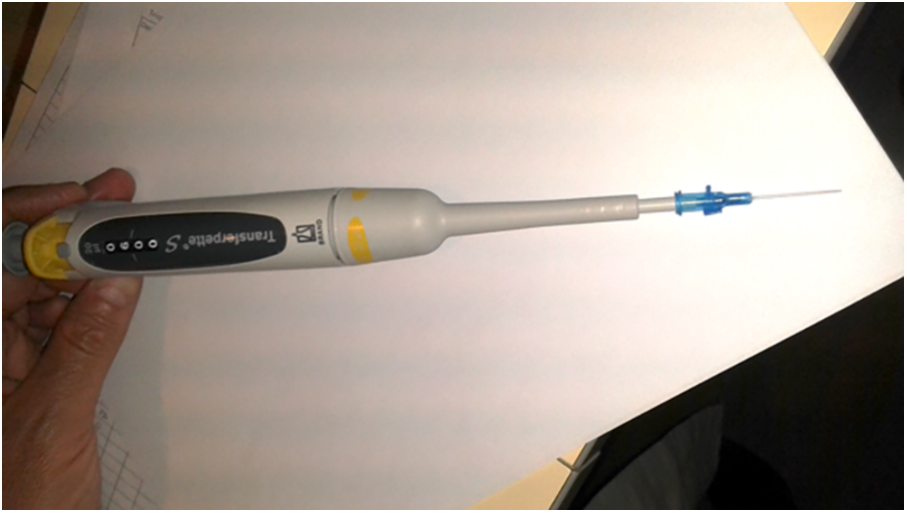
Variable volume pipette connected to a venous cannula used to administer propranolol eye micro-drops.

A more recent study confirmed the efficacy of propranolol eye drops at a concentration of 0.2% without significant adverse effects (113). To reduce the percentage of the drug absorbed through the conjunctival and nasal vessels and to increase the safety profile of the study, the infants received micro-drops administered using a variable volume pipette (Figure 1). However, the main limitation of this approach is the lack of a randomized controlled trial confirming its efficacy. Therefore, the topical strategy is yet a promising approach but not yet the treatment of choice.

Studies about long-term outcomes deriving from the use of propranolol are not available. A single study conducted by Filippi et al. (114) investigated the refractive errors of newborns with ROP treated with propranolol. Propranolol does not seem to directly affect the refractive errors; however, considering that the refractive outcome worsens as ROP stages advance, we can postulate that treatment with propranolol might indirectly improve the refractive outcome by reducing the progression of ROP.

## 9 Propranolol: mechanism of action

The way propranolol interferes with the molecular pathways involved in IH and ROP is not well understood, but many hypotheses are on the way. Propranolol is an unselective β-blocker, although previous studies have shown a preferential action on β2-ARs (79, 115, 116). The blockage of β-AR likely leads to inactivating effects both on canonical and non-canonical pathways downstream β2-AR.

**Figure 2:**
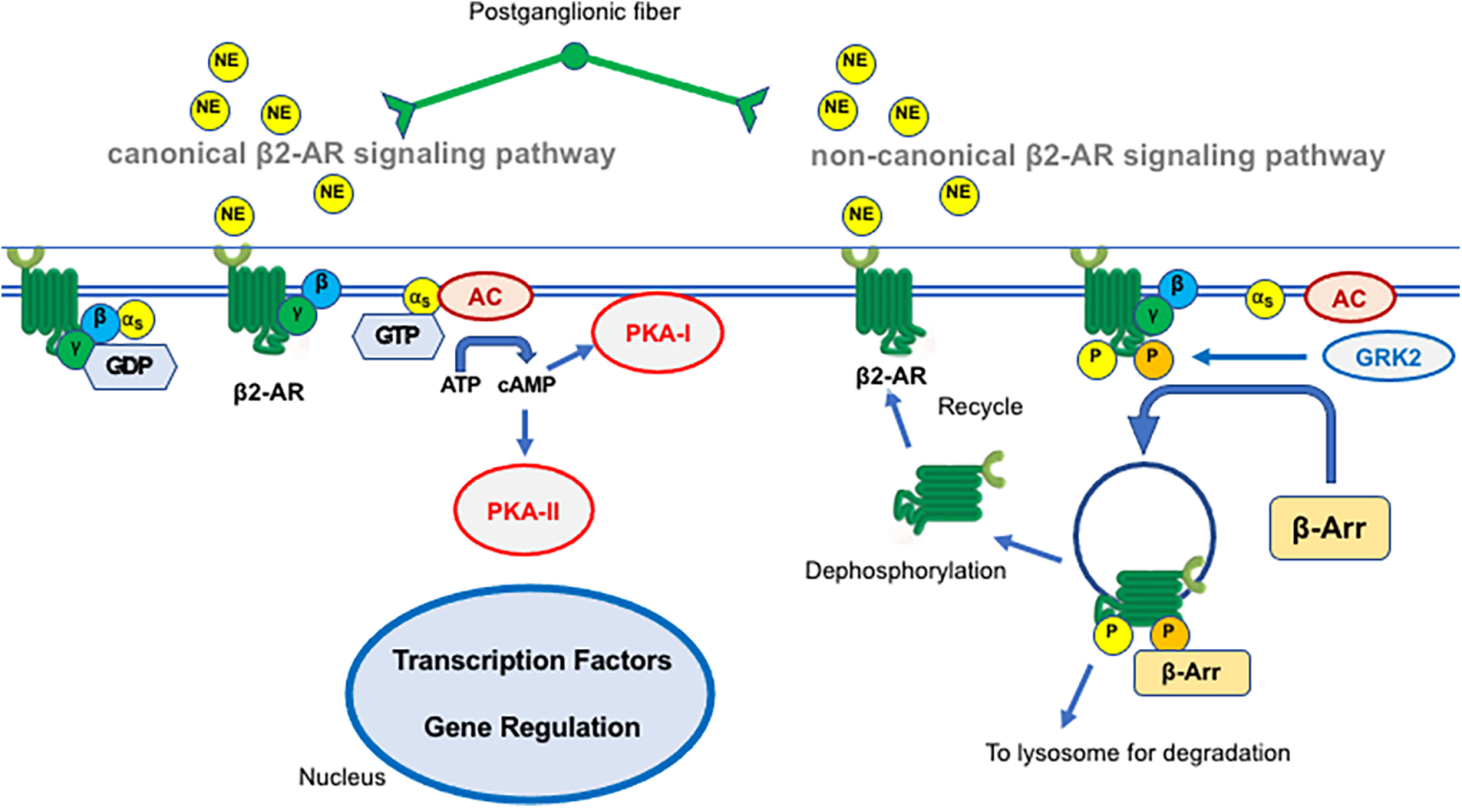
Schematic representation of the canonical (left) and non-canonical (right) β2-adrenergic receptor (β2-AR) signaling pathway. The mechanisms are described in the text. NE, norepinephrine; GTP, guanosine triphosphate; AC, adenylate cyclase; PKA, protein kinase A; GRK2, G protein-coupled receptor kinase 2; β-Arr, β-arrestin.

In the canonical pathway, the β2-AR couples dually to Gs and Gi proteins both converging on adenylate cyclase (AC). In the Gs pathway, increases in AC activity result in the elevation of intracellular cyclic AMP content and subsequently activation of protein kinase A (PKA). The majority of the downstream events stimulated by β2-AR agonists are therefore a consequence of the activation of PKA. PKA is composed of a regulatory subunit dimer, which is bound to a catalytic subunit. The regulatory subunits bind cAMP, an event that releases the catalytic subunits. The catalytic subunits, once released from the regulatory subunits, catalyze the transfer of ATP terminal phosphates to serine or threonine residues in target proteins. Phosphorylation of intracellular target proteins promotes the activity of transcription factors including HIF-1α. The final HIF-1α-associated player, VEGF, activates its receptor VEGFR2 to then reverberate on the PKA pathway ultimately leading to HIF-1α accumulation (Figure 2).

In the presence of sympathetic overstimulation, the β2-AR switches its coupling from Gs to Gi thus reversing the Gs-mediated generation of cAMP. Reduced PKA activity after β2-AR coupling to Gi blunts β1-AR signaling. Cross-talk between β1- and β2-AR is relevant to providing a negative feed-forward mechanism to sharpen the transient response of β1-AR in heart failure where Gi activation reduces the contractile response to β1-AR activation by norepinephrine upon sympathetic overactivation. At the vascular level, Gi activation switches from cAMP–PKA to mitogen-activated protein kinase (MAPK)/ERK pathways leading to endothelial nitric oxide synthase (eNOS) uncoupling and increased nitrosylation of eNOS that participate in endothelial dysfunction (116). Overall, propranolol exhibits an antiangiogenetic effect by inhibiting HIF-1α -induced VEGF expression, which is instead promoted by catecholaminergic overstimulation (117).

In the non-canonical pathway, activated β2-AR undergoes phosphorylation by several kinases of which G protein-coupled receptor kinase GRK2 is the prototype GRK for β2-AR desensitization. Following its phosphorylation, β2-AR becomes able to interact with members of the arrestin family, in particular with β-arrestin 2, thus triggering internalization and desensitization (Figure 2). In this respect, African American infants have a lower incidence of threshold ROP (61) and it has been speculated that this could be in part the consequence of a polymorphism of G protein-coupled receptor kinase mostly found in this ethnic group (118): this variant facilitates β-AR phosphorylation and thus desensitization, making then this population “genetically β-blocked” (119).

Interestingly, propranolol seems to exhibit its antiangiogenetic action irrespectively on β-AR blockade. Propranolol is a racemic mixture of R(+) and S(–) enantiomers and the R(+) enantiomer is completely devoid of β-blocker activity. In the case of IH, Overman et al. (120) demonstrated that propranolol inhibits HemSCs differentiation into hemangioma cells by disrupting the dimerization of SOX18, which is a transcriptional factor involved in endothelial cell differentiation and tumor-angiogenesis. Subsequently, Seebauer et al. (121) demonstrated that (R)+ enantiomers display this activity on HemSCs *in vivo* possibly interfering with SOX18 binding to chromatin. Moreover, propranolol inhibits the proliferation of both hemangioma endothelial and HemSCs, increasing the expression of adipogenesis-associated genes in stem cells (122). This data is confirmed by Li et al. (123), who demonstrated an increased transdifferentiation of HemSCs into adipocytes after treatment with propranolol, maybe acting on the PI3K pathway. How propranolol exerts all these effects has yet to be better clarified.

## 10 Conclusion

The unexpected demonstration that propranolol counteracts hypoxia-induced vascularization in IHs and ROP demonstrates that, in different anatomical regions, hypoxia promotes vascularization through the interaction between catecholamines and β-ARs. The use of propranolol therefore allows for decoupling the effects of hypoxia on the modulation of vascularization (36).

These observations open the way to a simple, non-invasive, economical treatment for ROP which aims to slow down the evolution of the disease without waiting for progression to the threshold of retinal detachment. The prospect of administering propranolol through ocular micro-drops makes this treatment particularly safe and presents it as the therapeutic opportunity of first choice.
